# Loss of a Conserved tRNA Anticodon Modification Perturbs Cellular Signaling

**DOI:** 10.1371/journal.pgen.1003675

**Published:** 2013-08-01

**Authors:** Boris Zinshteyn, Wendy V. Gilbert

**Affiliations:** Department of Biology, Massachusetts Institute of Technology, Cambridge, Massachusetts, United States of America; The University of North Carolina at Chapel Hill, United States of America

## Abstract

Transfer RNA (tRNA) modifications enhance the efficiency, specificity and fidelity of translation in all organisms. The anticodon modification mcm^5^s^2^U^34^ is required for normal growth and stress resistance in yeast; mutants lacking this modification have numerous phenotypes. Mutations in the homologous human genes are linked to neurological disease. The yeast phenotypes can be ameliorated by overexpression of specific tRNAs, suggesting that the modifications are necessary for efficient translation of specific codons. We determined the *in vivo* ribosome distributions at single codon resolution in yeast strains lacking mcm^5^s^2^U. We found accumulations at AAA, CAA, and GAA codons, suggesting that translation is slow when these codons are in the ribosomal A site, but these changes appeared too small to affect protein output. Instead, we observed activation of the *GCN4*-mediated stress response by a non-canonical pathway. Thus, loss of mcm^5^s^2^U causes global effects on gene expression due to perturbation of cellular signaling.

## Author Summary

Ribosomes translate the messages of the genetic code into functional proteins with the help of transfer RNAs (tRNAs), which carry a specific amino acid at one end and recognize three letters of the genetic code (a codon) with the other. tRNAs are subject to extensive chemical modifications, which are thought to enhance the efficiency, fidelity and specificity of translation. Many of these modifications are conserved across all domains of life, underscoring their biological importance. Despite intensive biochemical characterization, the physiological roles of most tRNA modifications are unknown. The tRNA modification mcm^5^s^2^U^34^ is required for normal growth and stress resistance in yeast; mutants lacking this modification have numerous phenotypes. Mutations in the homologous human genes are linked to neurological disease. The yeast phenotypes can be ameliorated by overexpression of specific tRNAs, suggesting that the modifications are necessary for efficient translation of specific codons. In order to test this model, we determined ribosome distributions at each codon in yeast strains lacking mcm^5^s^2^U. The changes we found appeared too small to affect protein output. Instead, we observed a non-canonical activation of a yeast stress response pathway. Thus, loss of mcm^5^s^2^U causes widespread perturbation of cellular signaling, independent of any codon-specific translation defects.

## Introduction

Transfer RNAs (tRNAs) from all domains of life contain numerous post-transcriptional modifications, many of which are highly conserved. These modifications enhance the efficiency, specificity and fidelity of translation [1]–[3]. In the budding yeast *Saccharomyces cerevisiae*, three tRNAs 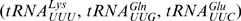 are modified by addition of 5-methoxycarbonylmethyl (mcm^5^) and 2-thio (s^2^) groups to uridine at the 5′ nucleotide of the tRNA anticodon (U34), resulting in an mcm^5^s^2^U nucleotide. The mcm^5^ s ^2^ U modification (MSUM) and many of the responsible modifying enzymes are conserved across eukaryotes, having been identified in fungi [4], [5], plants [6], worms [7] and mammals [8]. Despite widespread conservation, and extensive biochemical characterization, the physiological role of MSUM is unknown.

Genes required for MSUM are unusual among tRNA modification genes in the number and severity of their mutant phenotypes. Most yeast strains lacking tRNA modifications are viable and show no growth impairment [2], [3], but *S. cerevisiae* and *C. elegans* double mutants lacking both mcm^5^ and s^2^ are not viable [7], [9]. In yeast, single mutants lacking either mcm^5^ or s^2^ have numerous phenotypes including temperature sensitivity, various chemical stress sensitivities, exocytosis defects, and transcriptional defects [10], [11]. In *C. elegans*, mutants of the Elongator complex (comprised of *elp1* through *elp6*), which is required to produce the mcm^5^ modification, display neurological defects [7]. In humans, mutations in IBKAP, the *elp1* homolog, cause familial dysautonomia (FD) [12], and mutations in *elp4* are associated with Rolandic epilepsy [13].

The molecular connection between these cellular/organismal phenotypes and the lack of specific tRNA anticodon modifications is currently unknown. Loss of either mcm^5^ or s^2^ impairs reading of both Watson-Crick (VAA) and wobble (VAG) cognate codons by the modified tRNAs [14], [15], and chemical removal or modification of the s^2^ moiety leads to a reduction in the rate of tRNA charging *in vitro* [16], [17]. The MSUM phenotypes were originally attributed to a proposed role of the Elongator complex in transcriptional elongation [18] before its function in tRNA modification was discovered [4]. However, the phenotypes of yeast MSUM mutants, including the lethality in mutants lacking both mcm^5^ and s^2^, can be suppressed by overexpression of unmodified versions of two tRNAs that normally contain mcm^5^s^2^U – 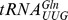 and 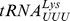 [11]. These observations indicate that at least a subset of the yeast cellular phenotypes are tied to tRNA function. It has been argued that loss of MSUM leads to codon-specific translation defects leading to insufficient protein production, either from many genes, or from a few genes required to carry out particular cellular processes or stress responses, but this hypothesis has not been directly tested.

In this study, we examined codon level ribosome distributions genome-wide using ribosome footprint profiling (Ribo-seq). We found that loss of mcm^5^ or s^2^ leads to slow translation elongation specifically at codons that Watson-Crick pair with MSUM tRNAs, but the magnitude of these changes appeared insufficient to affect protein output. Surprisingly, all of the MSUM strains showed gene expression signatures consistent with activation of the Gcn4p-mediated stress response pathway. We demonstrate that disruption of this pathway suppresses the MSUM mutant phenotypes independently of tRNA concentration.

## Results

### Ribosome Footprint Profiling Reveals Features of Translation for Specific Codons

**Figure 1:**
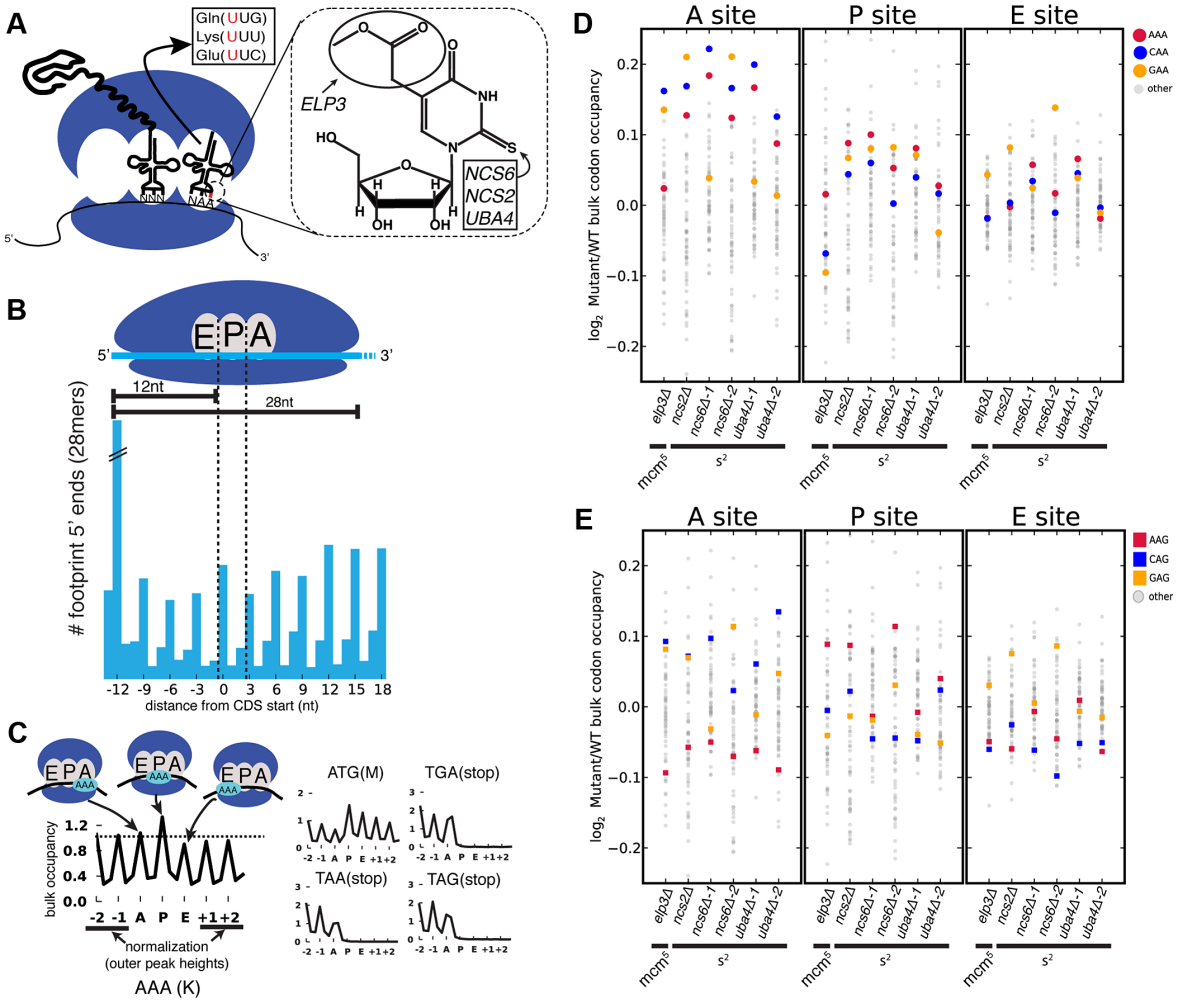
Genetic ablation of mcm^5^ or s^2^ leads to ribosome accumulation at specific codons. (**A**) (left) mcm^5^s^2^U is found at the 5′ nucleotide of the anticodon in three yeast tRNAs. (right) The structure of mcm^5^s^2^U, and the subset of modification genes whose mutants were profiled in this study are indicated. (**B**) (top) Anatomy of a ribosome footprint, with P-site offset for 28 mer reads indicated. (bottom) Metaplot of WT ribosome footprint reads summed across all start codons. The peak of upstream reads corresponds to ribosomes with start codons in their P site. The location of this peak is used to determine the location of A, P and E sites for each read length. (**C**) (left) Explanation of metacodon plots. Similar to panel B, all in-frame instances of a given codon in the genome are aligned, and the reads mapping around those positions are summed. The resulting plot is then offset by the P-site distance, and normalized to the average peak height of the outer sites (±1, ±2). The peak heights for each site are the bulk codon occupancies, a proxy for the amount of time the ribosome spends with a given codon in each site, compared to its neighbors. (right) ATG codons and stop codons display the expected distributions with this metric. All plots are from WT yeast. (**D** and **E**) Changes in bulk codon occupancy in MSUM mutants. Both plots are the same, with different codons highlighted. Independent biological replicates were done for *ncs6Δ* and *uba4Δ*. All mutants are compared to a WT sample prepared and processed simultaneously.

We set out to determine whether MSUM mutants display codon-specific translation defects. Translational activity genome-wide was determined using Ribo-seq, which consists of isolating and sequencing ribosome-protected mRNA fragments from RNase-treated whole-cell lysates [19]. This method reveals ribosome positions at single nucleotide resolution, and thus has the potential to identify translational defects affecting single codons [19], [20]. Wild type (WT) yeast, as well as strains lacking the s^2^ moiety (*ncs2Δ, ncs6Δ*, and *uba4Δ*), or mcm^5^ (*elp3Δ*) (Figure 1A), were profiled by Ribo-seq, as well as RNA-seq. To assess the impact of these modifications on translation, the ribosome dwell time at specific codons was determined as follows. The positions of the A, P and E site codons within ribosome footprints of various lengths (25–31 nt) were determined by examining the 5′ ends of footprints mapping to start codons, where initiating ribosomes are expected to contain start codons in their P sites (Figure 1B) [21]. Next, to determine the genome-wide average ribosome dwell time for a given codon (Figure 1C, left), all instances of that codon in the genome were aligned, and 5′ ends of reads mapping to the surrounding positions (Figure 1C) were summed (see Materials and Methods). The resulting metacodon plots show the relative number of ribosome footprints, and thus the relative amount of time the ribosome spends at each position, as the codon moves through the A, P and E sites. Codon identity is not expected to affect translation from the outer sites (±1, ±2), so the entire plot was normalized to the height of these peaks. The height of each peak is the bulk occupancy for that codon in that ribosomal site, similar to a previously described metric [20]. The metacodon distributions for ATG and stop codons indicated that the reads were properly assigned to the ribosomal sites (Figure 1C, right). We observed distinct and reproducible patterns of ribosome density for different codons in WT yeast (Figure 1C, S1A,B), consistent with the single-nucleotide resolution of this technique.

The metacodon plots of WT yeast provided insights into the determinants of translation rate for specific codons. Notably, all four proline codons spent over 2-fold more time in the P site than the average codon, while glycine codons spent ∼40–50% more time in the A site (Figure S1C). This effect was additive for Pro-Gly pairs in the P and A sites, but not if the codon order was reversed (Figure S1D), indicating that the effects of Pro and Gly were specific to the P and A sites, respectively. This proline effect is reminiscent of the proline/glycine pausing recently discovered in bacteria lacking elongation factor P [22]–[24]. The observed effects were consistent with *in vitro* data which showed that peptidyl transfer can be rate limiting for A-site glycine and proline codon translation at physiological pH [25], and that proline induces particularly slow peptide bond formation when it is at the carboxyl terminus of the growing peptide chain [26] (Figure S1E). These results suggest that peptidyl transfer is rate limiting for certain Pro and Gly codons in yeast cells as well.

Experiments in recombinant systems have led to the strong expectation that translation times for codons should be inversely proportional to the concentrations of their cognate tRNAs [27], [28]. To investigate potential sources of the distinctive metacodon distributions we observed, we performed unsupervised hierarchical clustering on them (Figure S2A). This analysis clustered many codons together based on their encoded amino acid or the first two nucleotides of the codon. Notably, codons did not cluster by tRNA adaptation index (tAI), a proxy for cognate tRNA abundance [27]. More directly, the bulk occupancies did not show a negative correlation with tAI in the A site (Figure S2B). There was also no correlation of codon occupancy with tRNA abundance measurements, genomic copy number, or a more recent codon usage metric which accounts for tRNA competition [29] (data not shown). These results demonstrate that translation rates for particular yeast codons are not determined by the cellular concentrations of their cognate tRNAs, consistent with findings from Ribo-seq experiments in mice and bacteria [30], [31] and from protein synthesis reporters (containing codon repeats) in yeast [32].

### Loss of MSUM Genes Reduces Translation Rate at AAA, CAA, GAA Codons

Having established the ability to detect differences in the translation of different codons, we next examined changes in codon-specific translation in the MSUM strains. Bulk occupancy for each codon in each ribosomal site (the height of the peaks in the metacodon plots) was determined for each mutant. All of the strains lacking the s^2^ modification showed increases in ribosome density corresponding to CAA and AAA in the A site, while the *elp3Δ* strain showed an increase in the CAA and GAA codons (Figure 1D). The magnitude of the changes was largest when the affected codon was found in the ribosomal A-site. The magnitude and direction of change for the GAA codon was variable between mutants lacking the same modification, and even between biological replicates (Figure 1D), indicative of some underlying biological or technical noise in this measurement. Nonetheless, in all but one replicate, the largest increases in each mutant were for codons decoded by Watson-Crick pairing with MSUM tRNAs.

### mcm^5^s^2^U Is Not Required for Wobble Decoding of AAG, CAG, and GAG Codons *In Vivo*

**Figure 2:**
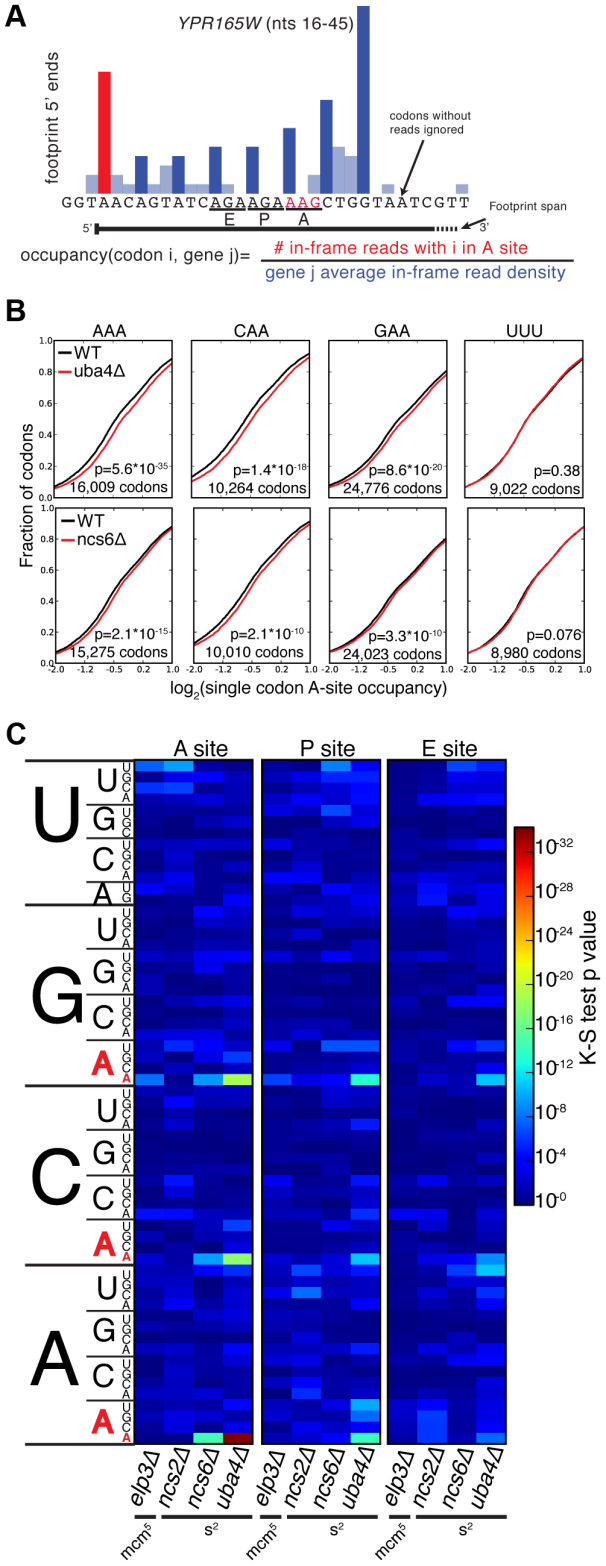
A single-codon occupancy metric shows that ribosome footprint accumulations at AAA, CAA, and GAA are statistically significant. (**A**) Description of the single codon occupancy metric. The occupancy for a given codon in a given site is the number of in-frame reads for that codon in that site, compared to the average in-frame read density for the parent gene. (**B**) Cumulative distributions of single-codon occupancy for select codons in *ncs6Δ* and *uba4Δ*. (**C**) Heatmap of K-S test p-values for all sense codons in all mutants. For *ncs6Δ* and *uba4Δ*, mutant and WT replicates were pooled to improve the accuracy of the metric.

MSUM is necessary for wobble decoding of G-ending codons in strains that lack other cognate tRNAs [14], but it is not clear whether the modified tRNAs contribute to decoding in the WT state where these other tRNAs are present. In our datasets AAG, CAG, and GAG codons showed smaller increases in bulk occupancy (and some net decreases) compared to their A-ending counterparts, suggesting that MSUM is mainly required for translation of VAA codons (Figure 1E). In order to assess the statistical significance of these changes, a metric for ribosome dwell time at individual codons was developed (Figure 2A). This metric normalizes the read counts at a particular codon by the mean read density of the open reading frame that contains it. The genome-wide distributions for all instances of each codon were compared between mutant and WT strains using the K-S test (Figure 2B, C). Due to the noise inherent in read sampling, many codons showed statistically significant changes. However, the VAA codons had p values many orders of magnitude smaller than all other codons, particularly in the *ncs6Δ* and *uba4Δ* datasets, which were from pooled biological replicates (Figure 2C). The pooled datasets provided data for approximately twice as many codons and may have averaged out biological and technical noise. Consistent with our analysis of bulk codon occupancy, the effect of MSUM loss was strongest in the A site for all 3 VAA codons. We did not see a corresponding statistical significance for the VAG codons (Figure 2C), indicating that mcm^5^s^2^U does not significantly contribute to the decoding of these codons *in vivo*. This result does not contradict previous evidence that the modifications are required for translation of VAG codons by wobble pairing [14], but indicates that tRNAs_UUB_ contribute minimally to the translation of VAG codons *in vivo*, where tRNAs_CUB_ with Watson-Crick complementarity are available.

### The Elongation Defects in MSUM Strains Appear Insufficient to Affect Protein Levels

**Figure 3:**
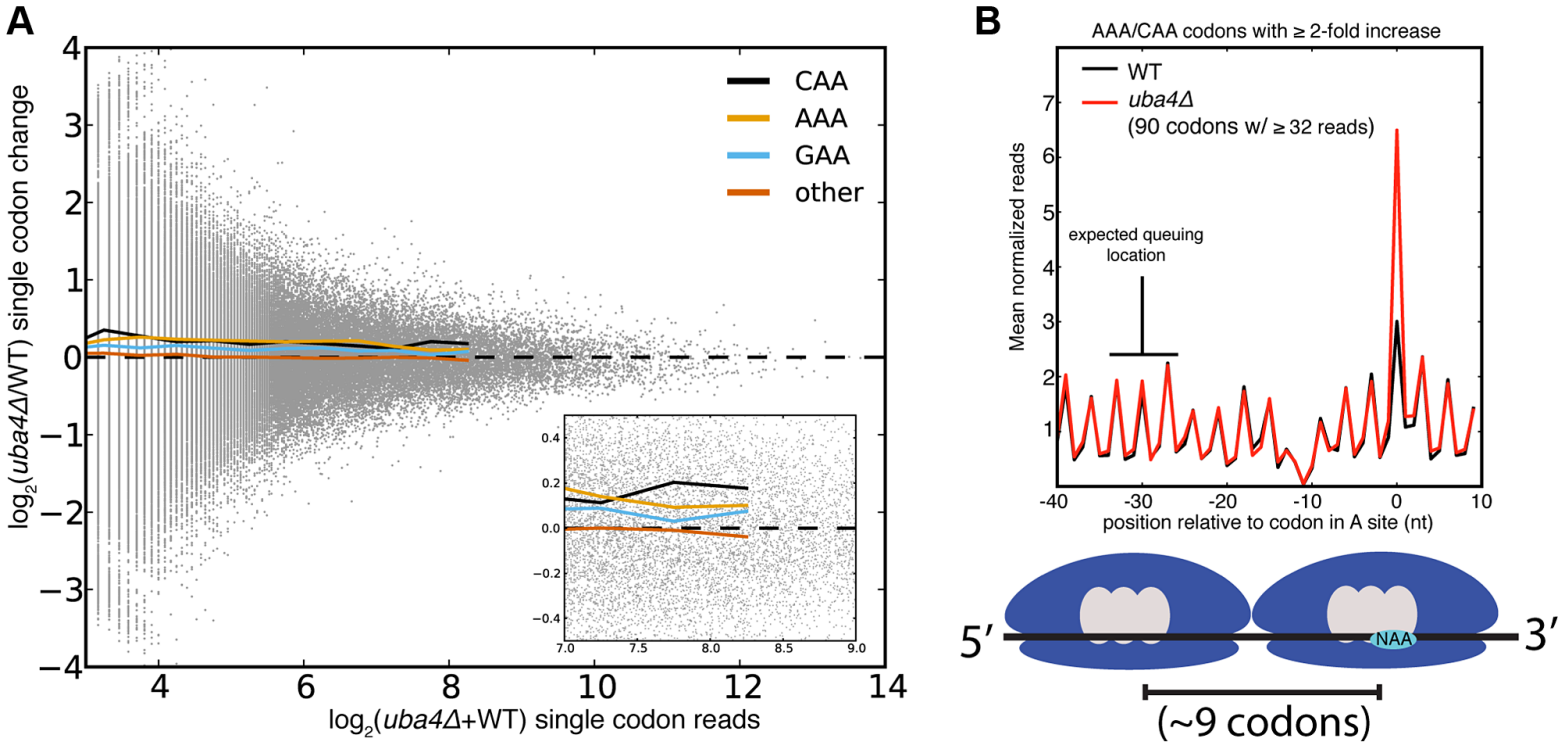
Single codon occupancy changes may be insufficient to affect protein output. (**A**) Fold changes for all single codons in *uba4Δ* are plotted against their read density in grey. Colored lines are the mean fold changes for the specified codons over read-coverage bins of width 0.2 (log_2_ scaled). “Other” is a pool of all non-VAA codons. (**B**) Metaplot of ribosome footprint density around all AAA and CAA codons with ≥2-fold change in *uba4Δ*, and ≥32 reads in both datasets. Reads at each position were normalized by the total number of reads for the parent gene, and averaged across all host genes that overlap that position. The plot is offset such that 0 corresponds to having the codon in the A site. The expected location of a ribosome queuing event is indicated, and a diagram of such an event is shown below. The dip in ribosome footprint density at −10 is a computational artifact, due to an inability to determine read lengths of poly-adenylated fragments when they end in one or more adenosines.

Despite the statistical significance of the increased ribosome dwell times at VAA codons in MSUM mutants, the magnitude of the changes does not seem to be large enough to generally affect protein output. Initiation, not elongation, is the rate-limiting step of eukaryotic translation in most circumstances [33], [34], and the mean ribosome density is only 1 per 164 nts [35]. Given this sparse spacing of ribosomes on yeast mRNAs, transcripts with mean ribosome density would require an elongation delay greater than the average translation time of 50 codons in order for an MSUM mutation to make elongation rate limiting. The most densely populated messages would require a 20-fold elongation delay. The average bulk increase observed for VAA codons was less than 17% (Figure 1D), and the largest confidently assigned (≥32 reads) single-codon change was less than 5-fold (Figure 3A, S3A). In the event of an elongation delay long enough to affect protein output, ribosome queuing should occur behind AAA and CAA codons with increased occupancy. However, no queuing was observed (Figure 3B, S3B). Codons with more read coverage display smaller changes than codons with low read coverage, indicating that the range of this metric is not being limited by sequencing depth (Figure 3A, S3A). We also did not observe increased ribosome density at stretches of 2 or more VAA codons (data not shown). These results were consistent with the polysome gradient profiles of the MSUM strains, which were indistinguishable from WT (data not shown), indicating that translation elongation in bulk was unaffected.

### The GCN4-Mediated Stress Response Is Activated in MSUM Strains

**Figure 4:**
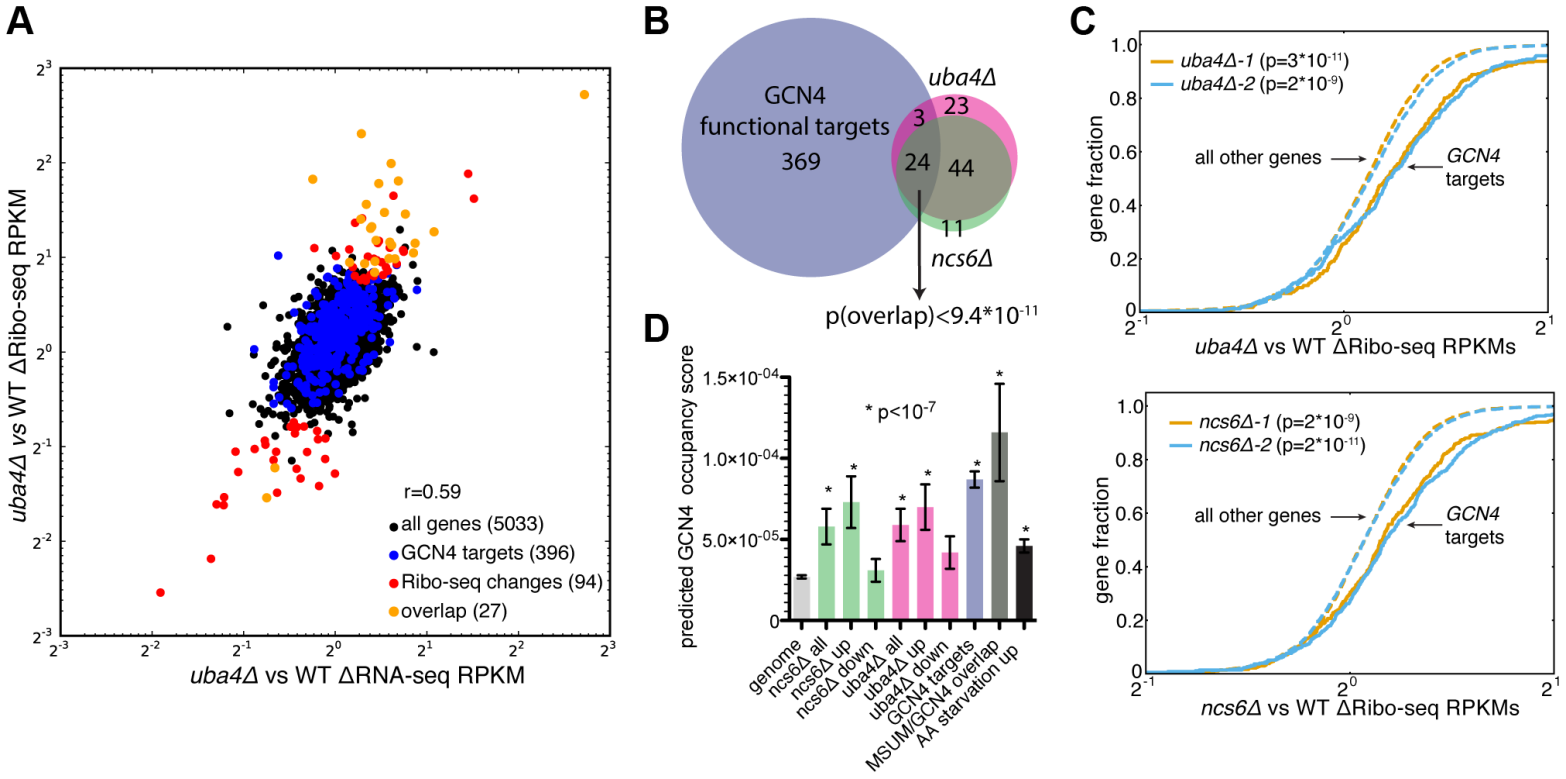
MSUM strains show the gene-expression signatures of *GCN4* activation. (**A**) Comparison of RNA-seq and Ribo-seq RPKM changes in *uba4Δ*. *GCN4* targets and statistically significant Ribo-seq changes are indicated. Values are the means of 2 biological replicates. (**B**) Venn diagram of overlap between *GCN4* functional targets (blue) and significant Ribo-seq RPKM changes in *uba4Δ* (pink) and *ncs6Δ* (green). The significance of the overlap was computed using the hypergeometric distribution. (**C**) Cumulative distribution plots of fold Ribo-seq changes for *GCN4* targets (solid lines) compared to all other genes (dashed lines) in *uba4Δ* (top) and *ncs6Δ.* P values are from a KS test of *GCN4* targets against the rest of the genome. (**D**) Mean±SEM of predicted Gcn4p occupancy for groups of genes from panel B and figure S5, as determined by high-throughput *in vitro* binding assays [40]. Bars are colored to match groups in panel B. P values are from t-tests comparing the indicated gene set against all genes in the genome.

In search of an alternative explanation for MSUM mutant phenotypes, we examined global ribosome footprint densities and transcript levels for perturbations in the MSUM mutant strains. Consistent with previous reports [19], [36], gene expression values from Ribo-seq were highly reproducible (Figure S4A). Furthermore, all of the mutant strains showed similar RNA-seq and Ribo-seq changes when compared to WT strains (Figure S4B,C), indicating that these gene expression changes are likely to be downstream of a common defect. Replicate data for *ncs6Δ* and *uba4Δ* enabled us to assess the significance of particular changes using counting statistics [37]. This analysis identified a set of genes with significant changes in ribosome footprint density, which were largely shared between *ncs6Δ* and *uba4Δ* (Figure 4A,4B,S4D). The changes in ribosome footprint density were correlated with changes in transcript levels (r = 0.59 for *ncs6Δ*, 0.64 for *uba4Δ*), indicating that these gene expression changes were largely due to changes in the mRNA pool (Figure 4A, 4D). Intriguingly, a significant fraction (24/68) of the affected genes are known targets of the *GCN4* transcription factor [38] (Figure 4A,4B,S4D). To investigate the specificity of the observed induction of *GCN4* targets in MSUM mutants, we examined the behavior of *GCN4* targets in 1,924 yeast microarray studies using data from the SPELL curated yeast microarray compendium. This compendium includes experiments sampling a broad range of environmental and genetic perturbations [39]. We determined the significance of overlap between *GCN4* targets and the set of upregulated (≥2-fold) genes in each of these 1,924 datasets. Notably, the overlap between *GCN4* targets and induced genes in MSUM strains was more statistically significant than the overlap between *GCN4* targets and induced genes in 82% of the SPELL datasets. The datasets with a higher degree of overlap consisted mostly (at least 276/343) of gene deletions and stress conditions in which *GCN4* is known to play a role (e.g. heat, nutritional perturbation, osmotic stress and DNA damage) (Table S4, data not shown). Furthermore, *GCN4* targets as a whole showed increased ribosome footprint density in all MSUM strains (Figure 4C, data not shown). We further confirmed this enrichment for functional *GCN4* targets by examining the predicted Gcn4p binding affinity of the promoters for the affected genes [40]. The promoter regions of the upregulated genes were enriched for Gcn4p binding motifs (Figure 4D). Using the same sets of upregulated genes from the SPELL compendium as above, less than 6% of these upregulated gene sets had a mean predicted Gcn4p occupancy greater than the genes upregulated in the MSUM strains (Table S4). Thus, GCN4 target genes are transcriptionally upregulated in all MSUM strains.

**Figure 5:**
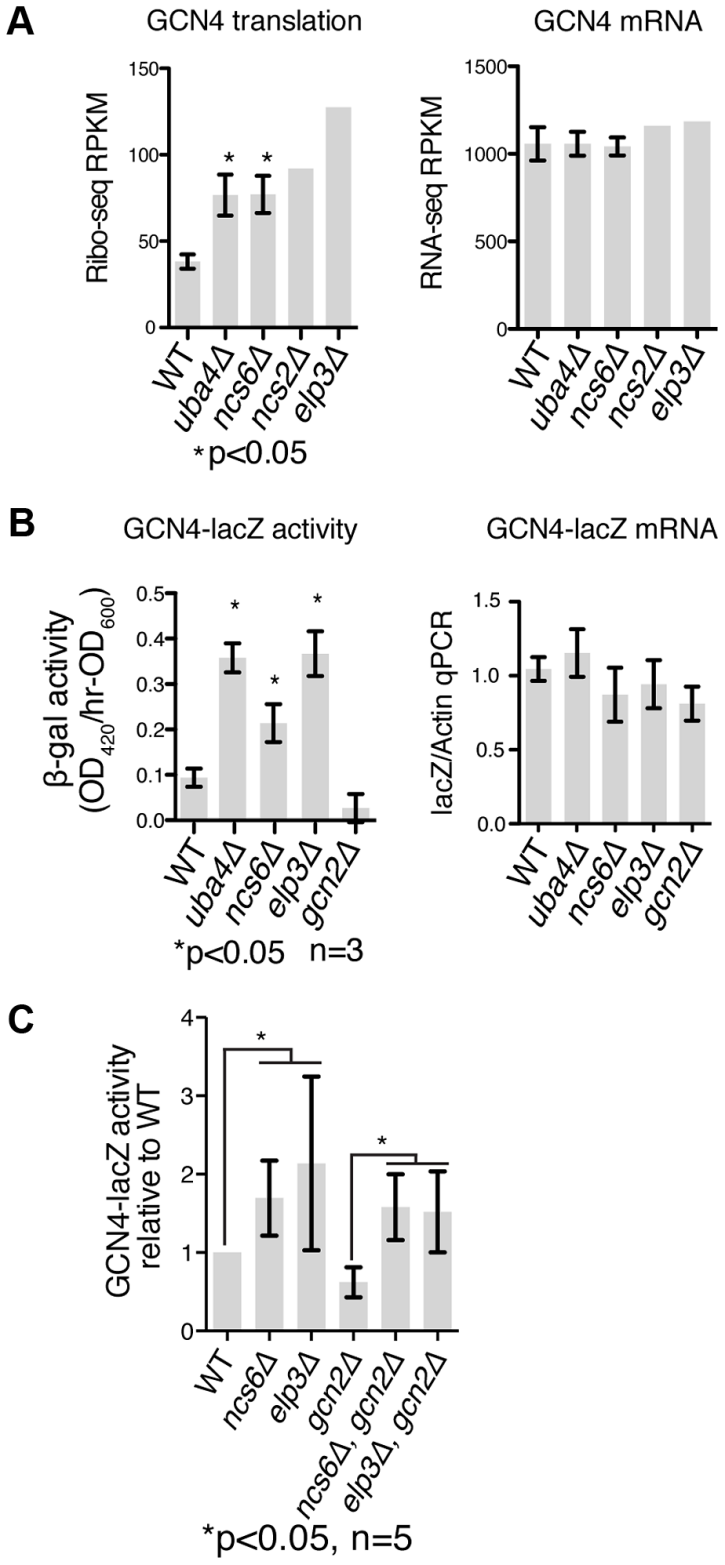
*GCN4* is induced independently of *GCN2* in MSUM strains. (**A**) Ribo-seq and RNA-seq RPKMs for the *GCN4* open reading frame. Standard deviations are indicated for strains with replicate data. (**B**) The indicated strains were transformed with a reporter containing the promoter and transcript leader of *GCN4* fused to *lacZ*. *LacZ* activity and mRNA levels were measured in log phase after overnight growth in YPD. (**C**) *LacZ* assays were performed as in panel B, with the addition of double mutant strains. P values are for t-test against WT unless otherwise indicated.

To provide context for these gene expression changes, the same analyses were performed on Ribo-seq data from yeast subjected to amino acid (AA) starvation, a well-characterized *GCN4*-inducing condition [19]. 20 minutes of amino acid starvation leads to a 4-fold increase in ribosome footprints on the *GCN4* ORF (data not shown). A larger number of genes displayed changes in AA starvation compared to MSUM ablation, and *GCN4* targets as a group had larger fold changes (median 2.0-fold induction vs. 1.2 and 1.1-fold for *uba4Δ* and *ncs6Δ* respectively). (Figure S5A, S5B). However, a smaller fraction of the significantly changing genes are GCN4 targets (13% in AA-starved cells, vs 29% and 30% for *uba4Δ* and *ncs6Δ* respectively) (Figure 5B, S5C). Furthermore, the starvation-induced genes had a smaller enrichment for predicted Gcn4p occupancy in their promoters compared to genes upregulated in the MSUM strains (Figure 5D). The limited induction of high-affinity Gcn4p targets in MSUM mutants is consistent with a weak but specific activation of the *GCN4* pathway.

### Induction of *GCN4* Occurs Independently of *GCN2*

We next sought to identify the mechanism of *GCN4* pathway induction in MSUM strains. *GCN4* is known to be translationally regulated in response to a variety of insults, most notably by amino acid starvation [41]. Translational repression of *GCN4* is mediated by four upstream open reading frames (uORFs), which prevent ribosomes from initiating on the protein-coding ORF. Conditions that decrease the efficiency of re-initiation allow some ribosomes to scan through the uORFs and initiate at the *GCN4* ORF. All four MSUM mutants showed ∼2-fold translational upregulation of *GCN4*, as evidenced by increased ribosome footprint density in the ORF with no increase in mRNA levels (Figure 5A).

A reporter construct containing the transcript leader of *GCN4* fused to *lacZ* verified that the uORF-containing leader was sufficient to recapitulate the translational induction observed in MSUM strains (Figure 5B). The magnitude of this induction (2–4 fold) is consistent with a weak activation of the GCN pathway, as a 3 hr shift to SC-Ura, and a constitutive *GCN2* allele [42] induced GCN4-lacz 7-fold and 50-fold, respectively (data not shown). The best-characterized pathway of inducing *GCN4* involves the activation of the Gcn2p kinase by uncharged tRNA, leading to phosphorylation of eukaryotic initiation factor 2α (eIF2α) and reduced efficiency of initiation and re-initiation. We therefore tested the effect of *gcn2Δ* on *GCN4* induction by MSUM mutants. Surprisingly, *GCN4-lacZ* was still induced in MSUM strains lacking GCN2 (Figure 5C). In addition, basal eIF2α phosphorylation levels were not increased in the MSUM strains, consistent with a *GCN2*-independent mechanism (data not shown). Thus, GCN4 translational induction in MSUM strains occurs by a non-canonical pathway.

In addition to the canonical *GCN2*-dependent response, some tRNA charging and modification defects have been shown to cause induction of *GCN4* by a *GCN2*-independent mechanism [43]–[45]. MSUM mutations may affect charging. *In vitro* experiments have shown that loss of the s^2^ moiety of MSUM tRNAs reduces the efficiency of tRNA charging [16], [17], although steady state tRNA charging levels are unaltered in MSUM mutants [14]. We reasoned that a kinetic defect in tRNA charging could lead to compensatory increases in tRNA synthetase gene expression [46], which could suppress steady-state charging defects. We examined synthetase expression by unsupervised hierarchical clustering of mRNA abundance changes in all of the mutant strains. GlnRS, LysRS, GluRS and AspRS formed a cluster of increased expression in the MSUM mutants (Figure S6). Three of these synthetases (Gln, Lys, and Glu) have MSUM tRNAs as substrates. The specific upregulation of this set of tRNA synthetases, along with the global activation of *GCN4* targets, suggests that MSUM mutants have adjusted their cellular state to cope with the loss of the mcm^5^s^2^U modification (see Discussion).

### Disruption of the GCN Pathway Partially Suppresses Some MSUM Phenotypes

**Figure 6:**
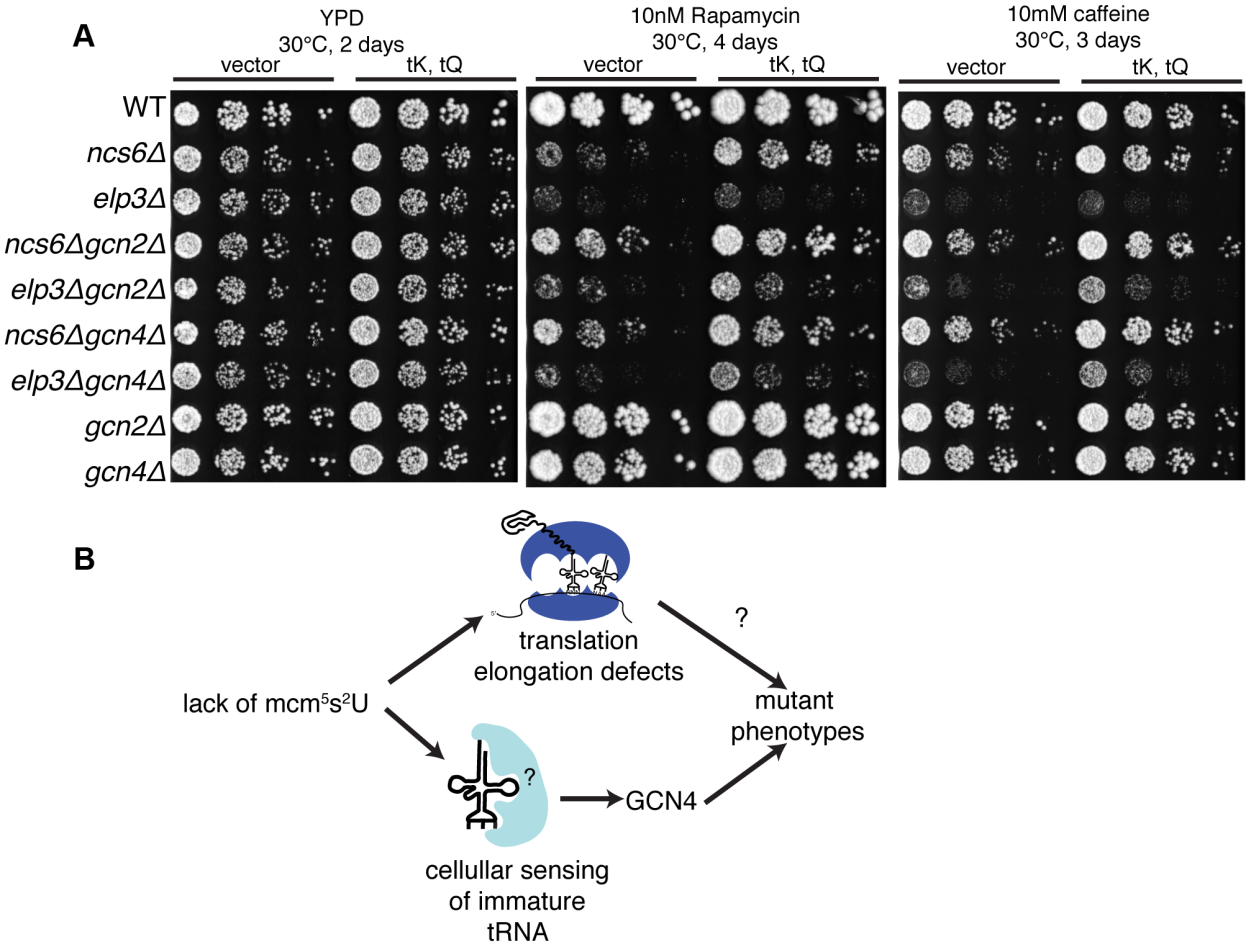
Disruption of the GCN pathway partially suppresses the stress sensitivity of MSUM strains, independently of tRNA overexpression. (**A**) Yeast was grown to saturation in selective media. 5-fold serial dilutions were spotted onto YPD containing the indicated drug, and grown at the indicated temperature. (**B**) The independent rescue of MSUM phenotypes by *gcnΔ* and hc-tRNA suggests that two independent pathways contribute to the mutant phenotypes.

To investigate the functional significance of *GCN4* misregulation in MSUM mutants, double mutants were constructed between *gcn2Δ* or *gcn4Δ* and *ncs6Δ* or *elp3Δ*, and tested for growth under conditions where MSUM mutants grow poorly. Under heat (40°C), caffeine and diamide stress, *gcnΔ*/MSUM double mutants showed some increase in growth compared to the single MSUM mutants (Figure 6A,S7A). On rapamycin, the suppression by *gcn* deletion was similar in magnitude to the suppression by high-copy (hc)-tRNA (Figure 6A). We did not observe any rescue of slow growth on YPD at 30°C with either GCN deletion or hc-tRNA expression (Figure 6A,S7B). Expressing hc-tRNA in the double mutant strains conferred additional resistance in all stress conditions, indicating that the *GCN* pathway contributes to the MSUM phenotypes independently of the pathway affected by hc-tRNA expression (Figure 6B).

## Discussion

MSUM tRNA modifications are conserved throughout eukarya and are required for organismal fitness in yeast, *C. elegans*, and humans. Due to the striking phenotypes of MSUM mutants, as well as the reported suppression by hc-tRNA [11], we expected to find large increases in ribosome density at codons decoded by MSUM tRNAs. We did detect increased ribosome density at VAA codons, and the largest effects of MSUM ablation occurred in the ribosomal A-site, the only site where tRNA binding, and thus concentration, is expected to play a role [21]. Thus, our analysis was capable of detecting codon-level translation defects in these mutants. However, the small magnitude of the observed effect makes it unlikely that protein output is generally affected. Additionally, suppression by hc-tRNA was incomplete in our hands, and the extent of both phenotypes and suppression varied between *elp3Δ* and *ncs6Δ* mutants when they were directly compared, as opposed to examined separately as in previous studies [11]. This suggests that MSUM genes may play additional roles in the cell, or create tRNA defects that are not suppressible by tRNA overexpression.

Overall, we found complex and varied patterns of ribosome density surrounding the different codons of the genetic code. These patterns appear to be determined not by cognate tRNA concentrations, but by intrinsic properties of aminoacyl tRNAs or peptidyl transfer kinetics, consistent with previous data showing that synonymous codon usage had little effect on protein output when mRNAs were expressed at physiological levels [28], [47]. This overall result is also consistent with the results of a systematic study of protein output from codon-repeat reporters [32]. Our data do not recapitulate all of the findings of that study, most likely because the reporters contained unnaturally long stretches of rare codons and were expressed at levels high enough to deplete the native tRNA pool. Furthermore, unlike reporter gene assays, Ribo-seq is able to detect changes in translation rate that are too small to be detected in an assay for protein output.

Since tRNA concentrations vary over an order of magnitude [27], yet had little effect on ribosome distributions at different codons, it is hard to understand how a ∼2–3 fold overexpression of hypomodified tRNA [48] could strongly affect the rate of ribosome movement. Our data do not rule out the possibility that one or more lowly expressed genes have elongation defects in MSUM mutants that are sufficient to reduce protein output. If so, there must be additional features that make codons in those genes unusually sensitive to the lack of the mcm^5^s^2^U modification. Indeed, loss of MSUM has been shown to cause a reduction in protein output in artificially sensitized conditions, such as the readthrough of stop codons by a suppressor tRNA [4], [49]. It is also possible that larger codon-specific translation defects were not manifest in our growth conditions, which would be consistent with the inability of hc-tRNA to rescue the slow growth of MSUM mutants on YPD. Our data also do not rule out the possibility that a slight increase in ribosome dwell time could lead to amino acid misincorporation [50], misfolding of the protein product [51], or degradation of the mRNA and/or protein by the mRNA surveillance machinery [52]. Further experiments are needed to understand the mechanism(s) of phenotypic suppression by hc-tRNAs.

The largest changes detected in the MSUM mutants were transcriptional effects consistent with activation of the *GCN4* pathway. The gene expression signature of *GCN4* induction was noticed previously in *elpΔ* mutants [10], and was attributed to the presumed role of Elongator in transcription. However, the similarity of the *elp3Δ* gene expression changes to those of *ncs6Δ*, *ncs2Δ* and *uba4Δ*, which have clear roles in an independent tRNA modification pathway [5], [53], [54], argues against this explanation. Instead, it appears that improperly modified tRNAs elicit a cellular stress response.

There is precedent for *GCN2*-independent activation of the *GCN4* pathway by perturbations of tRNAs. Nuclear aminoacylation of tRNAs facilitates export to the cytoplasm in yeast and Xenopus oocytes [55], [56], and disruption of this process can lead to nuclear accumulation of tRNA, as well as *GCN2*-independent *GCN4* induction [43], [44]. Loss of the s^2^ modification has been previously shown to reduce the rate of *in vitro* aminoacylation reactions for MSUM tRNAs [16], [17]. This charging defect could lead to nuclear accumulation of tRNA and the observed *GCN2*-independent induction of *GCN4*, despite the normal steady-state levels of charged tRNA in MSUM strains [14]. The apparent transcriptional upregulation of all three synthetases that recognize MSUM tRNAs may reflect a cellular response to such a defect in tRNA charging. Consistent with a role for the *GCN* pathway in mediating physiologically relevant signaling in response to loss of MSUM, deletion of *GCN2* or *GCN4* partially suppressed the phenotypes of MSUM strains.

The observation that *GCN* deletion suppresses MSUM phenotypes independently of the phenotypic suppression conferred by hc-tRNA suggests that there are at least two independent pathways contributing to the MSUM phenotypes. This may have implications for Elongator complex mutants in higher eukaryotes. In *C. elegans*, rescue of MSUM phenotypes by hc-tRNA has not been demonstrated. Furthermore, the translational effects reported in *C. elegans* MSUM strains [7] are more consistent with a global decrease in translation initiation, as might be expected in conditions leading to *GCN4* activation, than with codon-specific elongation defects. Such secondary effects on gene expression may also play a role in the neurological symptoms of patients with mutations in *elp* genes. Indeed, induced pluripotent stem cells from FD patients with hypomorphic alleles of *elp1* display numerous transcriptional changes during differentiation compared to controls [57]. It will be important to determine the extent to which tRNA-responsive signaling and transcriptional changes, in addition to codon-specific translation defects, contributes to the phenotypes of MSUM mutants in higher eukaryotes, and the severe and varied symptoms of FD patients.

## Materials and Methods

### Yeast Strains and Culture Conditions

All strains (Table S1) were in the s288c BY4742 background (*MATα his3Δ1 leu2Δ0 lys2Δ0 ura3Δ0*). MSUM and *GCN* deletions strains were constructed by PCR-mediated gene replacement as previously described [58]. All strains were grown in YPD (1% Yeast extract, 2% Peptone, 0.01% Adenine hemisulfate, 2% Dextrose) unless otherwise indicated. For growth assays with hc-tRNA plasmids, strains were grown in SC-Leu to maintain selection. Strains were then plated onto YPD.

### Ribo-seq and RNA-seq

Yeast strains were grown from an OD600 of ∼0.001–0.004 in aerated flasks at 30°C to mid-log phase (OD ∼0.7), treated with 0.1 mg/ml cycloheximide for 2 minutes, and harvested by centrifugation. Cells were lysed by vortexing with glass beads, and libraries were prepared essentially as described [19], [36]. For the WT-2, *elp3Δ*, *ncs2Δ*, *uba4Δ-2*, *ncs6Δ-2* libraries, triton was omitted until after lysis. For any analysis in which only 2 libraries are compared, the mutant was always compared to the WT sample processed identically. Sequencing data were deposited in the GEO database with the accession number GSE45366.

### Read Mapping and Positional Assignment

Data analysis was performed using custom Python and Bash scripts developed in-house, unless otherwise indicated. Reads were mapped based on their 5′ 21 nt using Bowtie [59]. Reads were first mapped to *S. cerevisiae* rRNA, allowing up to 3 mismatches, and any mapping multiplicity. Any reads mapping to rRNA were discarded. Reads were then mapped to the *S. cerevisiae* genome downloaded from the saccharomyces genome database (SGD) on 5/26/2010, allowing up to 3 mismatches and requiring unique mapping. Read lengths were determined by comparing the original read sequence to the genomic sequence. Reads for which the beginning of the *in vitro* poly-A tail coincides with a genomic A have ambiguous length, and were excluded from length-specific analyses. Open reading frame (ORF) annotations downloaded from SGD were used to produce mappings of reads relative to the start codon for each ORF, which were used for all downstream calculations. For all codon-level analyses, reads of each length were processed separately, and 5′ end mapping locations were subsequently pooled, and shifted 5′ with the appropriate offsets (25:0, 26:0, 27:0, 28:0, 29:-1, 30:-1, 31:-2, negative numbers imply a 3′ shift) to put them in frame with 28 mer reads. When computing RPKMs (reads per kilobase of ORF sequence per million ORF reads) and read counts for each ORF, an unsplit pool of reads was used. The ORF positions are defined from 12 nt upstream of the start codon to 14 nt upstream of the stop codon. The first 8 codons of each ORF were excluded from all gene expression calculations to exclude possible artifacts from cycloheximide incubation.

### Metacodon Plots and Bulk Occupancy Calculations

The value of position i in the metacodon vector for codon NNN is computed as follows:
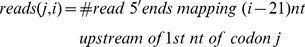
Where the 21 nt offset is the 28 mer P-site offset (12 nt) plus the distance from the p-site to the first nt in the metacodon plot.
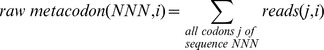
The normalized metacodon vector is computed by normalizing to the peak heights of the outer sites:
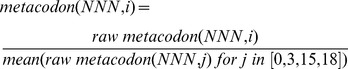
The mapping of metacodon peaks to ribosomal sites is: (0:-2, 3:-1, 6:A, 9:P, 12:E,15:+1,18:+2). For Figure S1D, the summation is performed over all codon positions for the given amino-acid pair, using the position of the first nucleotide of the first codon in the pair.

### Single Codon Occupancy Metric

The single codon occupancy for codon i in gene j in ribosomal site k is computed as:
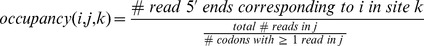
For both the numerator and denominator, only in-frame reads (those whose 5′ ends fall a multiple of 3 from the first nt of the site) were counted, and the first 4 codons, as well as codons with no in-frame reads were excluded.

### Hierarchical Clustering

For Figure S2, the normalized metacodon vectors for each codon were used as inputs for cluster 3.0 [60]. Codons were clustered using spearman correlation and single linkage. Heatmaps were generated using Java Treeview [61]. The tAI column was not used for clustering, and was only added afterwards for comparison. For Figure S5, centroid linkage was used for clustering.

### Queuing Analysis

For each AAA and CAA codon with ≥2-fold increase, the reads at each surrounding position were normalized by the mean read density for the entire ORF. These values were summed relative to all of the codons analyzed, offset so that the 0 position corresponds to the codon in the A site, and the value at each position was divided by the total number of codons whose host gene overlapped the given position. A secondary ribosome pileup is expected to occur approximately one ribosome footprint width (∼28 nt) upstream of the slow codon. Due to the use of polyadenylation in library preparation, any read ending in an adenosine cannot be assigned a length, and is not included in this analysis. Because of this, there is a depletion of read density at ∼−10 nts, corresponding to reads that end with 1 or more adenosines.

### Gene Expression Analysis

Significant Ribo-seq changes were called using edgeR [37]. Significance was assessed using a Bonferroni-corrected p-value cutoff of 0.05. The significance of overlap with *GCN4* targets was assessed using the hypergeometric test, and the definition of target genes derived from Natarajan *et al* [38]. The background for the hypergeometric test was defined as the set of genes with confident expression values for all datasets (5034 genes for MSUM datasets, 2780 for amino acid starvation).

### β-galactosidase Assays

Starter cultures containing the GCN4-lacZ reporter plasmid (Table S2) were grown to saturation in SC-URA, then diluted into YPAD and grown in conditions identical to the Ribo-seq samples. At an OD600 of 0.7–0.8, 1 ml aliquots each were taken for qPCR and β-galactosidase assays, spun down, media aspirated, and frozen. Pellets were resuspended in Z buffer and permeabilized as previously described [62]. Cell suspensions were transferred in triplicate to a transparent 96-well plate, and 1/5 volume of 4 mg/ml ONPG was added. OD420 was measured every minute for 1 hour in a Bio-Tek synergy HT plate reader. β-galactosidase activity was defined as the slope of the linear portion of the OD420 vs. time graph, normalized by the OD600 of the culture at harvest.

### Quantitative RNA Analysis

RNA was purified from yeast pellets as described [63]. Reverse transcription and quantitative PCR was performed using Avian Myeloblastosis Virus Reverse Trancriptase (AMV-RT; Promega) and real-time reagents (Invitrogen) according to manufacturer's instructions using a Roche Lightcycler 480. See Table S3 for gene-specific primer sequences.

### Automated Liquid Growth Assays

Liquid growth assays were carried out as previously described [64], except that saturated selective media starter cultures were diluted to an OD of 0.01 in YPD, then diluted 20-fold in YPD to a final volume of 100 µl.

## Supporting Information

Figure S1 Metacodon plots provide information on translation kinetics at the codon level. (**A**) Full set of metacodon plots, with superimposed WT replicates. (**B**) Reproducibility of bulk codon occupancy metric. Spearman correlations are indicated. (**C**) Details of metacodon plots for Gly and Pro. (**D**) Metacodon plots for Pro-Gly and Gly-Pro pairs. (**E**) Model for Pro and Gly metacodon plots. Peptidyl transfer is slow when Pro is in the P site, or Gly is in the A site, possibly making peptidyl-transfer rate-limiting for translocation, especially for Pro-Gly pairs.
(PDF)

Figure S2 Codon occupancy is not determined by codon adaptation. (**A**) Unsupervised hierarchical clustering of WT metacodon plots. Codons for the same amino acid that cluster together have been colored. The tRNA adaptation index (tAI) for each codon is indicated in red. The tAI is a proxy for cognate tRNA abundance. (**B**) Correlations between WT codon occupancy and tAI for codons in each ribosome site.
(PDF)

Figure S3 Single codon occupancy changes and queuing analysis for *ncs6Δ.* (**A**) Fold changes for all single codons in *uba4Δ* are plotted against their read density in grey. Colored lines are the mean fold changes for the specified codons over read-coverage bins of width 0.2 (log_2_ scaled). “Other” is a pool of all non-VAA codons. (**B**) Metaplot of ribosome footprint density around all AAA and CAA codons with ≥2-fold change in *uba4Δ*, and ≥32 reads between both datasets. Reads at each position were normalized by the total number of reads for the parent gene, and averaged across all host genes that overlap that position. The plot is offset such that 0 corresponds to having the codon in the A site. The expected location of a ribosome queuing event is indicated, and a diagram of such an event is shown below. The dip in ribosome footprint density at -10 is a computational artifact, due to an inability to determine read lengths of poly-adenylated fragments when they end in one or more Adenosines.
(PDF)

Figure S4 All MSUM mutants show similar gene expression changes compared to WT. (**A**) Reproducibility of Ribo-seq data. (**B**) Comparison of RNA-seq RPKM changes in mutant libraries. (**C**) Comparison of Ribo-seq RPKM changes in mutant libraries. Pearson r^2^ are presented in B-D. (**D**) Comparison of RNA-seq and Ribo-seq RPKM changes in *uba4Δ*, as in Figure 4A.
(PDF)

Figure S5 Amino acid starvation causes a stronger but less specific activation of GCN4 targets than MSUM ablation. (**A**) Comparison of RNA-seq and Ribo-seq RPKM changes in amino acid (AA) starved yeast (data from [19]). *GCN4* targets and statistically significant Ribo-seq changes are indicated. Values are the means of 2 biological replicates. (**B**) Venn diagram of overlap between *GCN4* functional targets (blue) and significant Ribo-seq changes upon AA starvation. The significance of the overlap was computed using the hypergeometric distribution. (**C**) Cumulative distribution plots of fold Ribo-seq changes for *GCN4* targets (solid line) compared to all other genes (dashed line). P values are from a KS test of *GCN4* targets against the rest of the genome.
(PDF)

Figure S6 Aminoacyl-tRNA synthetases for MSUM tRNAs show a coordinated mRNA upregulation in MSUM strains. Clustering of mRNA RPKM changes in MSUM strains clusters glnRS, lysRS, gluRS together. It is not clear why AspRS should be affected, but it has a unique regulatory mechanism [65], and clusters apart from the other synthetases in large scale microarray studies (data not shown).
(PDF)

Figure S7 Effects of GCN disruption or hc-tRNA on MSUM phenotypes. (**A**) Strains tested for growth in additional stress conditions. Yeast were grown to saturation in selective media. 5-fold serial dilutions were spotted onto YPD containing the indicated drug, and grown at the indicated temperature. (**B**) Doubling times for strains grown in liquid media. The means of two biological replicates, each with four technical replicates, is presented. The error bars indicate the propagated standard deviation of these measurements.
(PDF)

Table S1 Yeast strains used in this study.
(DOC)

Table S2 Plasmids used in this study.
(DOC)

Table S3 qPCR primers used in this study.
(DOC)

Table S4 Overlap of *GCN4* targets with transcriptional changes in SPELL datasets.
(TSV)
