# Supplementary figures and images for: Loss of a Conserved tRNA Anticodon Modification Perturbs Cellular Signaling
Source: PLoS Genet. 2013 Aug 1;9(8):e1003675. doi: 10.1371/journal.pgen.1003675 (PMC3731203; doi:10.1371/journal.pgen.1003675)

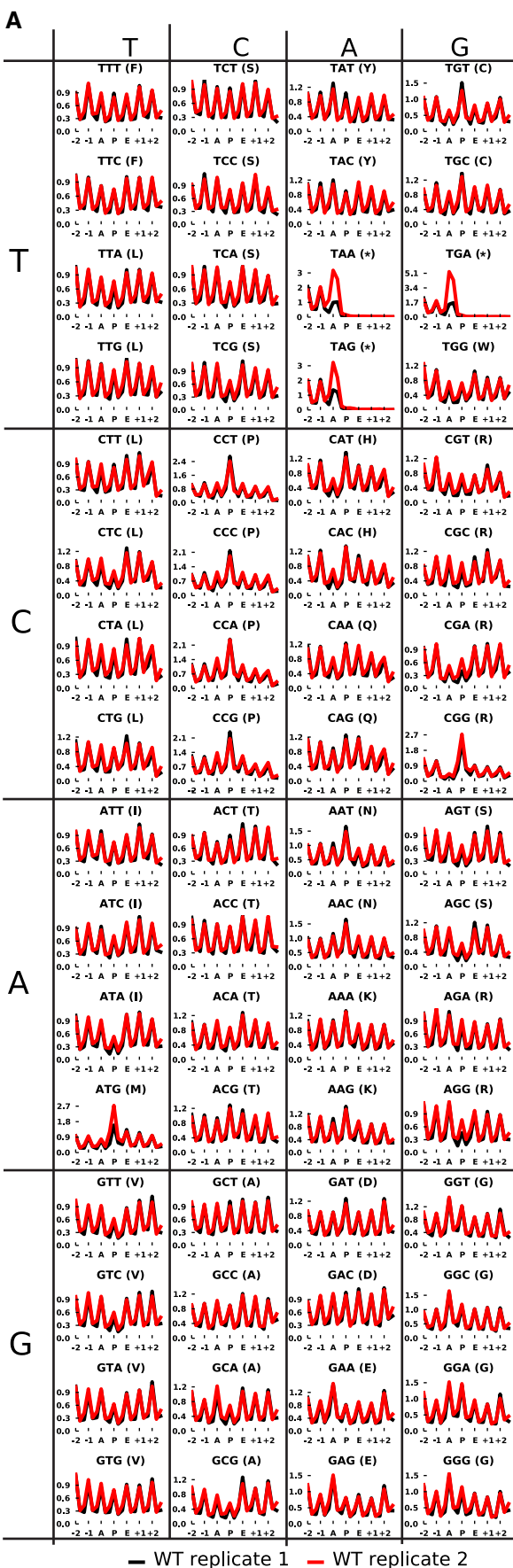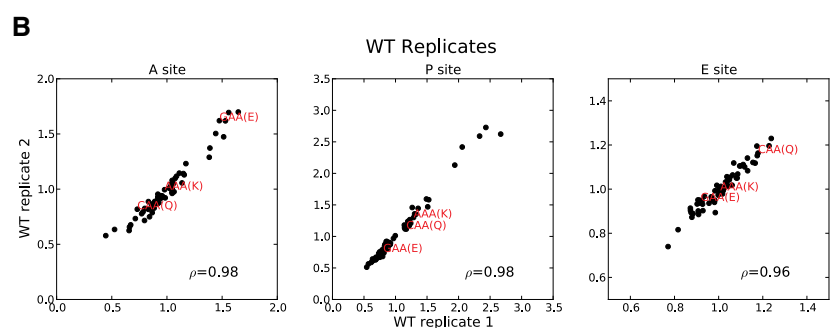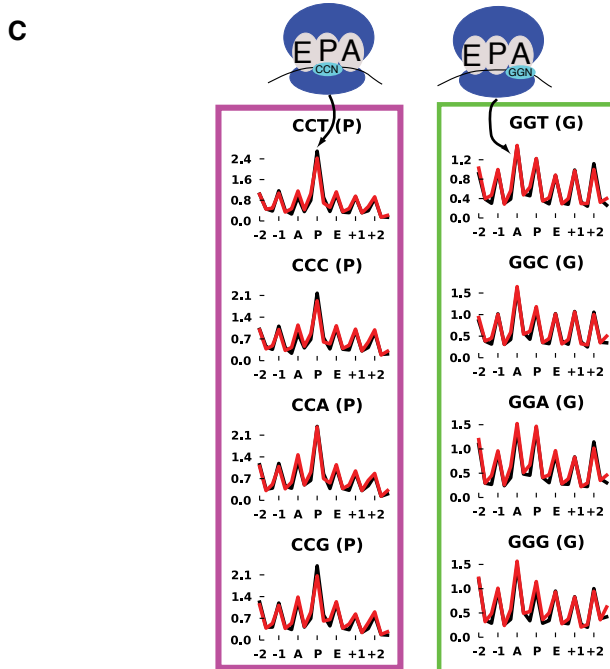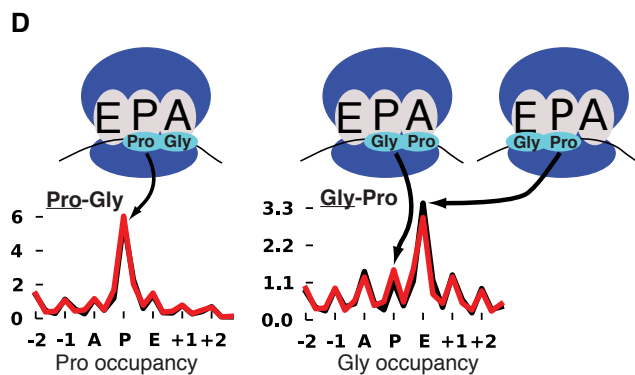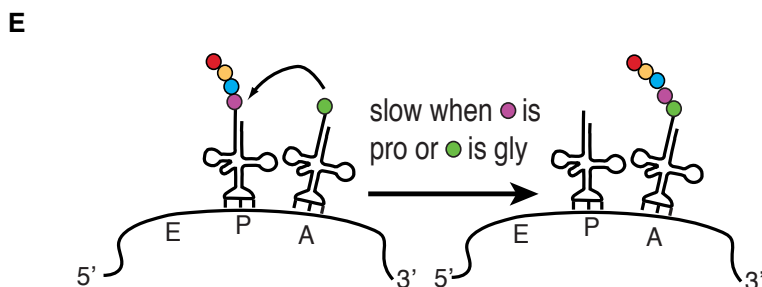

Supplement: Figure S1 — Metacodon plots provide information on translation kinetics at the codon level. (A) Full set of metacodon plots, with superimposed WT replicates. (B) Reproducibility of bulk codon occupancy metric. Spearman correlations are indicated. (C) Details of metacodon plots for Gly and Pro. (D) Metacodon plots for Pro-Gly and Gly-Pro pairs. (E) Model for Pro and Gly metacodon plots. Peptidyl transfer is slow when Pro is in the P site, or Gly is in the A site, possibly making peptidyl-transfer rate-limiting for translocation, especially for Pro-Gly pairs. (PDF) [file pgen.1003675.s001.pdf]

**A**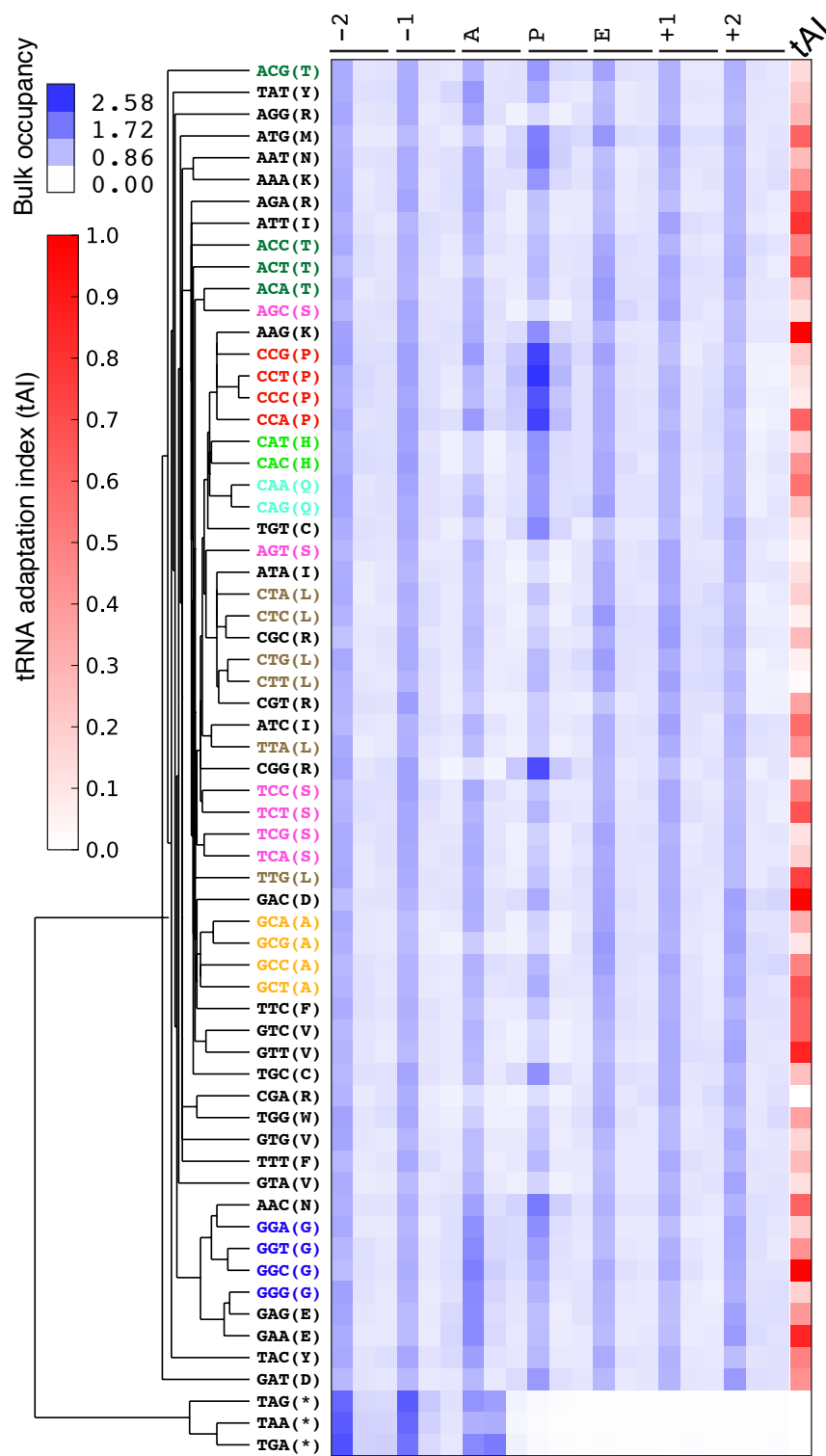**B**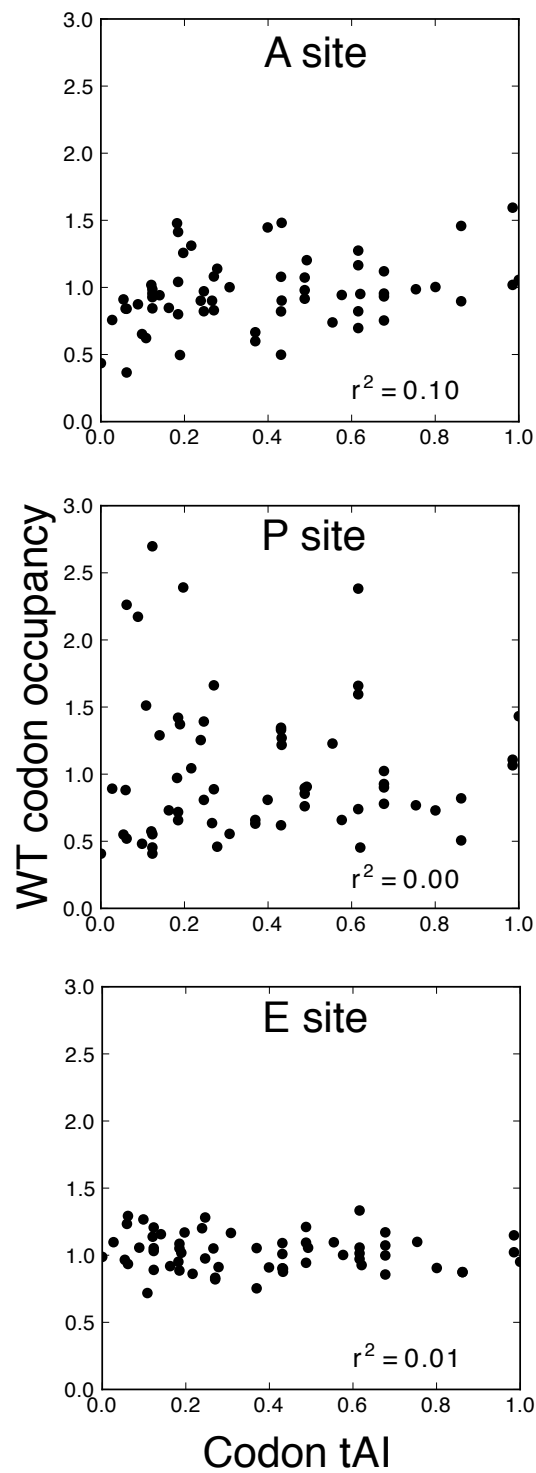

Supplement: Figure S2 — Codon occupancy is not determined by codon adaptation. (A) Unsupervised hierarchical clustering of WT metacodon plots. Codons for the same amino acid that cluster together have been colored. The tRNA adaptation index (tAI) for each codon is indicated in red. The tAI is a proxy for cognate tRNA abundance. (B) Correlations between WT codon occupancy and tAI for codons in each ribosome site. (PDF) [file pgen.1003675.s002.pdf]

**A**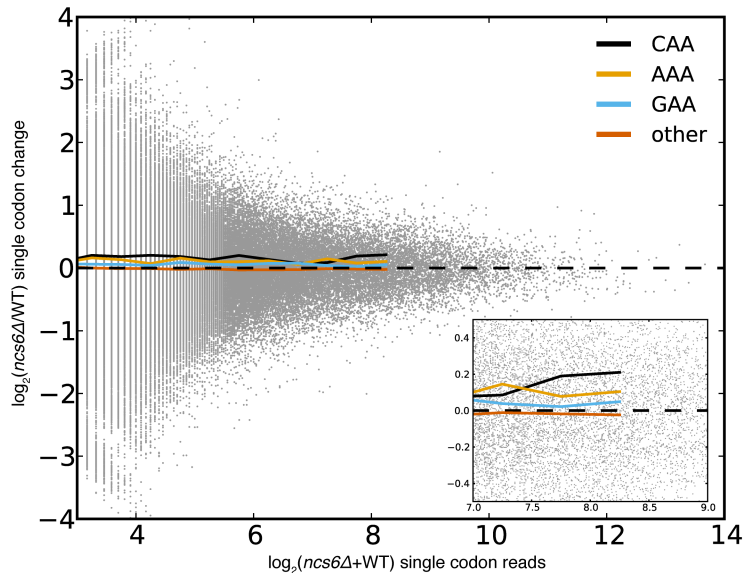**B**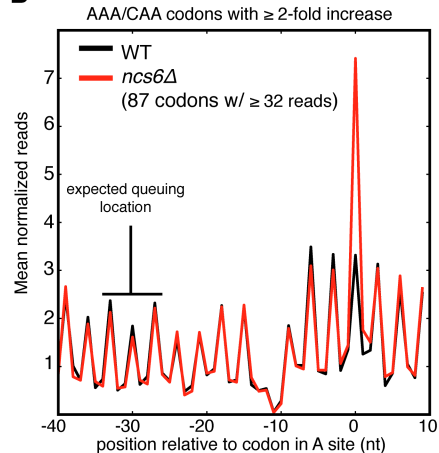

Supplement: Figure S3 — Single codon occupancy changes and queuing analysis for ncs6Δ. (A) Fold changes for all single codons in uba4Δ are plotted against their read density in grey. Colored lines are the mean fold changes for the specified codons over read-coverage bins of width 0.2 (log2 scaled). “Other” is a pool of all non-VAA codons. (B) Metaplot of ribosome footprint density around all AAA and CAA codons with ≥2-fold change in uba4Δ, and ≥32 reads between both datasets. Reads at each position were normalized by the total number of reads for the parent gene, and averaged across all host genes that overlap that position. The plot is offset such that 0 corresponds to having the codon in the A site. The expected location of a ribosome queuing event is indicated, and a diagram of such an event is shown below. The dip in ribosome footprint density at -10 is a computational artifact, due to an inability to determine read lengths of poly-adenylated fragments when they end in one or more Adenosines. (PDF) [file pgen.1003675.s003.pdf]

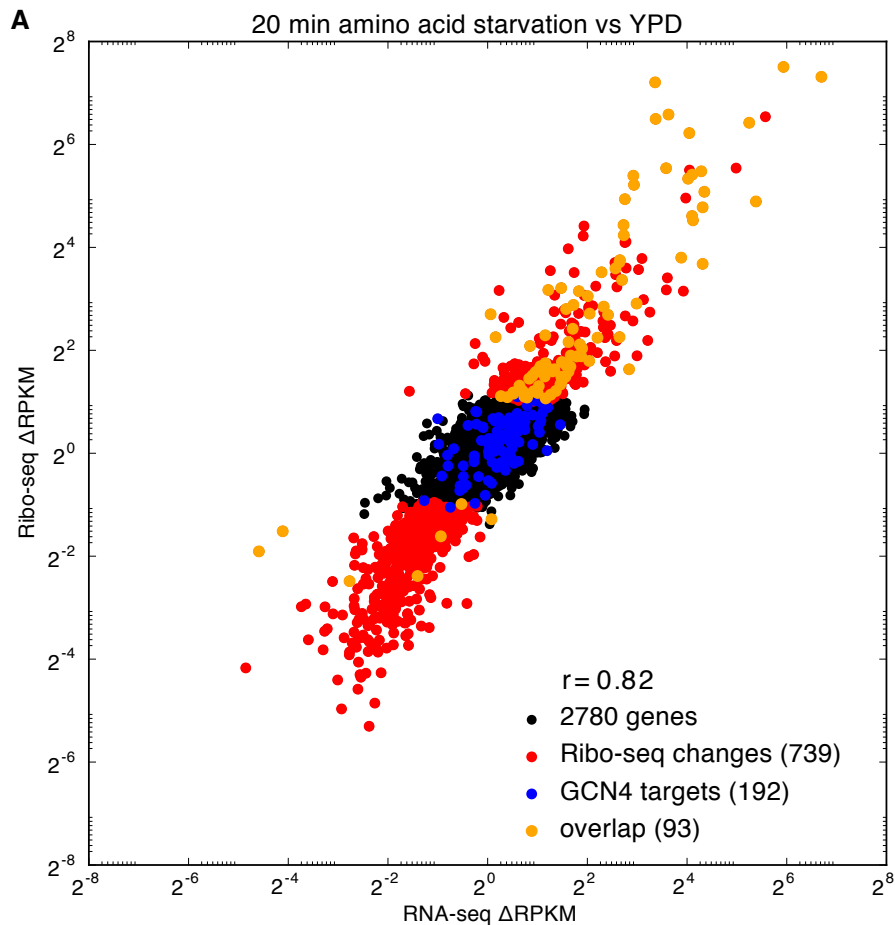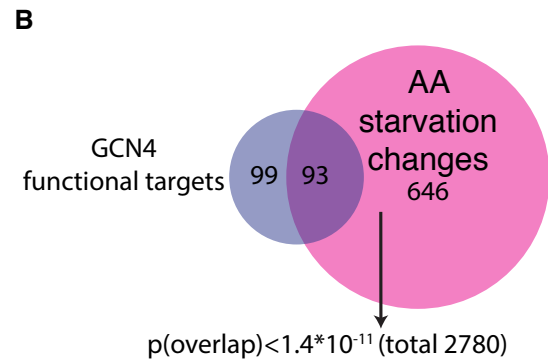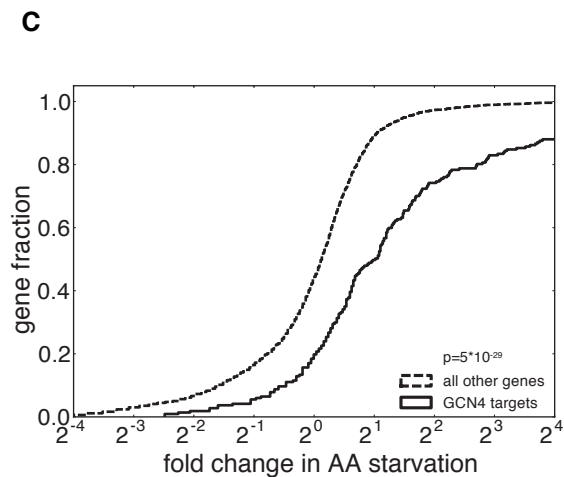

Supplement: Figure S5 — Amino acid starvation causes a stronger but less specific activation of GCN4 targets than MSUM ablation. (A) Comparison of RNA-seq and Ribo-seq RPKM changes in amino acid (AA) starved yeast (data from [19]). GCN4 targets and statistically significant Ribo-seq changes are indicated. Values are the means of 2 biological replicates. (B) Venn diagram of overlap between GCN4 functional targets (blue) and significant Ribo-seq changes upon AA starvation. The significance of the overlap was computed using the hypergeometric distribution. (C) Cumulative distribution plots of fold Ribo-seq changes for GCN4 targets (solid line) compared to all other genes (dashed line). P values are from a KS test of GCN4 targets against the rest of the genome. (PDF) [file pgen.1003675.s005.pdf]

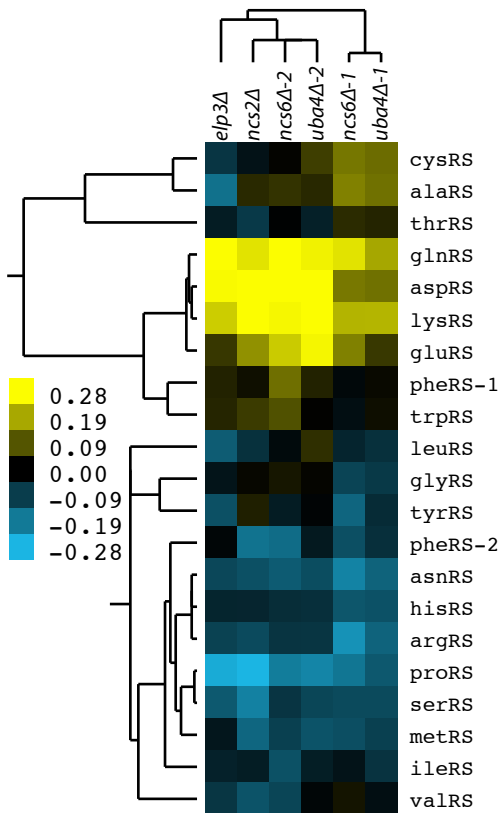

$\log_2$  mRNA fold change

Supplement: Figure S6 — Aminoacyl-tRNA synthetases for MSUM tRNAs show a coordinated mRNA upregulation in MSUM strains. Clustering of mRNA RPKM changes in MSUM strains clusters glnRS, lysRS, gluRS together. It is not clear why AspRS should be affected, but it has a unique regulatory mechanism [65], and clusters apart from the other synthetases in large scale microarray studies (data not shown). (PDF) [file pgen.1003675.s006.pdf]

**A**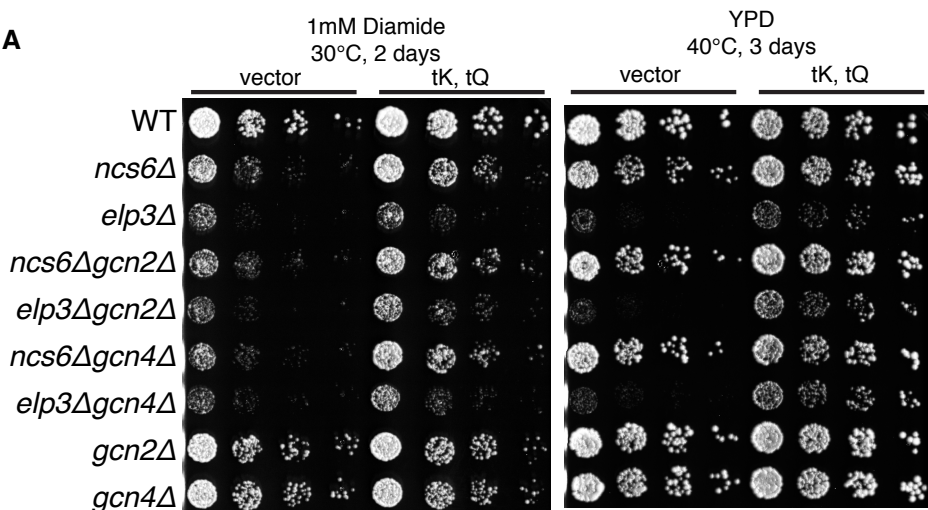**B**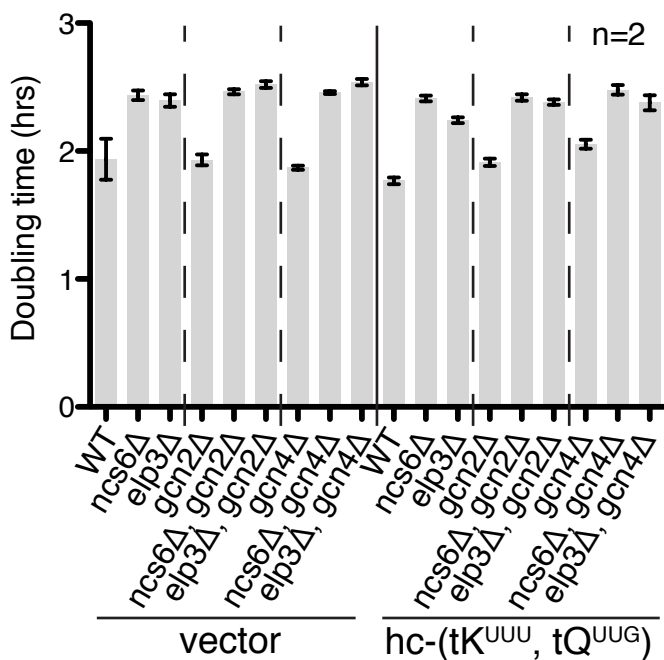

Supplement: Figure S7 — Effects of GCN disruption or hc-tRNA on MSUM phenotypes. (A) Strains tested for growth in additional stress conditions. Yeast were grown to saturation in selective media. 5-fold serial dilutions were spotted onto YPD containing the indicated drug, and grown at the indicated temperature. (B) Doubling times for strains grown in liquid media. The means of two biological replicates, each with four technical replicates, is presented. The error bars indicate the propagated standard deviation of these measurements. (PDF) [file pgen.1003675.s007.pdf]
