# Supplementary material for: Loss of a Conserved tRNA Anticodon Modification Perturbs Cellular Signaling
Source: PLoS Genet. 2013 Aug 1;9(8):e1003675. doi: 10.1371/journal.pgen.1003675 (PMC3731203; doi:10.1371/journal.pgen.1003675)
Supplement: Table S1 — Yeast strains used in this study. (DOC) [file pgen.1003675.s008.doc]

**Supplementary Table 1: Yeast strains used in this study**

| **Strain** | **Genotype** | **Plasmid** | **Source** |
| --- | --- | --- | --- |
| YWG11 | *MATα his3∆1 leu2∆0 lys2∆0 ura3∆0* |  | BY4742 |
| YWG269 | *MATα his3∆1 leu2∆0 lys2∆0 ura3∆0 ncs6::kan* |  | This study |
| YWG271 | *MATα his3∆1 leu2∆0 lys2∆0 ura3∆0 uba4::kan* |  | This study |
| YWG382 | *MATα his3∆1 leu2∆0 lys2∆0 ura3∆0 elp3::kan* |  | This study |
| YWG385 | *MATα his3∆1 leu2∆0 lys2∆0 ura3∆0 ncs6::kan* |  | This study |
| YWG386 | *MATα his3∆1 leu2∆0 lys2∆0 ura3∆0 ncs2::kan* |  | This study |
| YWG545 | *MATα his3∆1 leu2∆0 lys2∆0 ura3∆0* | p180 | This study |
| YWG547 | *MATα his3∆1 leu2∆0 lys2∆0 ura3∆0 ncs6::kan* | p180 | This study |
| YWG557 | *MATα his3∆1 leu2∆0 lys2∆0 ura3∆0 elp3::kan* | p180 | This study |
| YWG654 | *MATα his3∆1 leu2∆0 lys2∆0 ura3∆0 gcn2::kan* | p180 | This study |
| YWG1003 | *MATα his3∆1 leu2∆0 lys2∆0 ura3∆0 elp3::kan gcn2::nat* | p180 | This study |
| YWG1004 | *MATα his3∆1 leu2∆0 lys2∆0 ura3∆0 ncs6::kan gcn2::nat* | p180 | This study |
| YWG560 | *MATα leu2-3,112 ura3-52 ino1 GCN2c-516* | p180 | Ramirez *et al*. MCB, 1992 |
| YWG683 | *MATα his3∆1 leu2∆0 lys2∆0 ura3∆0* | pWG445 | This study |
| YWG684 | *MATα his3∆1 leu2∆0 lys2∆0 ura3∆0 ncs6::kan* | pWG445 | This study |
| YWG685 | *MATα his3∆1 leu2∆0 lys2∆0 ura3∆0 elp3::kan* | pWG445 | This study |
| YWG1005 | *MATα his3∆1 leu2∆0 lys2∆0 ura3∆0 ncs6::kan gcn2::nat* | pWG445 | This study |
| YWG1006 | *MATα his3∆1 leu2∆0 lys2∆0 ura3∆0 elp3::kan gcn2::nat* | pWG445 | This study |
| YWG1007 | *MATα his3∆1 leu2∆0 lys2∆0 ura3∆0 ncs6::kan gcn4::nat* | pWG445 | This study |
| YWG1008 | *MATα his3∆1 leu2∆0 lys2∆0 ura3∆0 elp3::kan gcn4::nat* | pWG445 | This study |
| YWG686 | *MATα his3∆1 leu2∆0 lys2∆0 ura3∆0 gcn2::nat* | pWG445 | This study |
| YWG699 | *MATα his3∆1 leu2∆0 lys2∆0 ura3∆0 gcn4::nat* | pWG445 | This study |
| YWG678 | *MATα his3∆1 leu2∆0 lys2∆0 ura3∆0* | pWG449 | This study |
| YWG679 | *MATα his3∆1 leu2∆0 lys2∆0 ura3∆0 ncs6::kan* | pWG449 | This study |
| YWG680 | *MATα his3∆1 leu2∆0 lys2∆0 ura3∆0 elp3::kan* | pWG449 | This study |
| YWG1009 | *MATα his3∆1 leu2∆0 lys2∆0 ura3∆0 ncs6::kan gcn2::nat* | pWG449 | This study |
| YWG1010 | *MATα his3∆1 leu2∆0 lys2∆0 ura3∆0 elp3::kan gcn2::nat* | pWG449 | This study |
| YWG1011 | *MATα his3∆1 leu2∆0 lys2∆0 ura3∆0 ncs6::kan gcn4::nat* | pWG449 | This study |
| YWG1012 | *MATα his3∆1 leu2∆0 lys2∆0 ura3∆0 elp3::kan gcn4::nat* | pWG449 | This study |
| YWG681 | *MATα his3∆1 leu2∆0 lys2∆0 ura3∆0 gcn2::nat* | pWG449 | This study |
| YWG704 | *MATα his3∆1 leu2∆0 lys2∆0 ura3∆0 gcn4::nat* | pWG449 | This study |
