# Supplementary material for: Loss of a Conserved tRNA Anticodon Modification Perturbs Cellular Signaling
Source: PLoS Genet. 2013 Aug 1;9(8):e1003675. doi: 10.1371/journal.pgen.1003675 (PMC3731203; doi:10.1371/journal.pgen.1003675)
Supplement: Table S2 — Plasmids used in this study. (DOC) [file pgen.1003675.s009.doc]

**Table S2: Plasmids used in this study**

| **Plasmid** | **Contents** | **Source** |
| --- | --- | --- |
| p180 | GCN4-lacZ, URA3, CEN4 | Alan Hinnebusch |
| pWG445 (pRS425) | LEU2, 2μ | Sebastian Leidel |
| pWG449 (pSZ64) | LEU2, tK(UUU), tQ(UUG), 2μ | Sebastian Leidel |
