# Supplementary material for: Loss of a Conserved tRNA Anticodon Modification Perturbs Cellular Signaling
Source: PLoS Genet. 2013 Aug 1;9(8):e1003675. doi: 10.1371/journal.pgen.1003675 (PMC3731203; doi:10.1371/journal.pgen.1003675)
Supplement: Table S3 — qPCR primers used in this study. (DOC) [file pgen.1003675.s010.doc]

**Table S3: qPCR primers used in this study**

| **Primer** | **sequence** |
| --- | --- |
| oBZ47-lacZ_F | GAAAGCTGGCTACAGGAAGG |
| oBZ48-lacZ_R | GTTGCACCACAGATGAAACG |
| oTC10-actin_F | TTCTGAGGTTGCTGCTTTGG |
| oTC11-actin_R | CTTGGTGTCTTGGTCTACCG |
