# Supplementary material for: Loss of a Conserved tRNA Anticodon Modification Perturbs Cellular Signaling
Source: PLoS Genet. 2013 Aug 1;9(8):e1003675. doi: 10.1371/journal.pgen.1003675 (PMC3731203; doi:10.1371/journal.pgen.1003675)
Supplement: Figure S4 — All MSUM mutants show similar gene expression changes compared to WT. (A) Reproducibility of Ribo-seq data. (B) Comparison of RNA-seq RPKM changes in mutant libraries. (C) Comparison of Ribo-seq RPKM changes in mutant libraries. Pearson r2 are presented in B-D. (D) Comparison of RNA-seq and Ribo-seq RPKM changes in uba4Δ, as in Figure 4A. (PDF) [file pgen.1003675.s004.pdf]

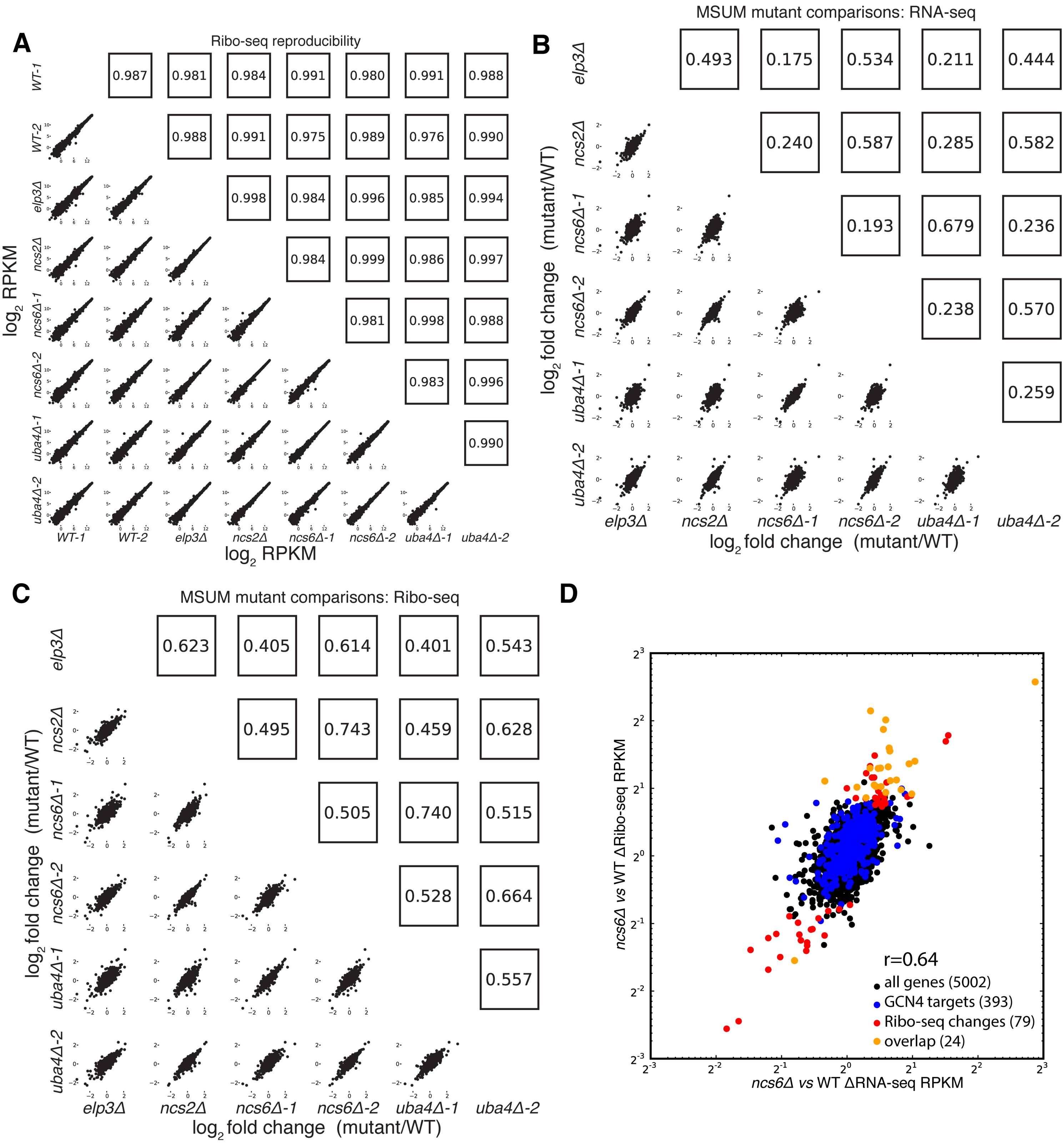

A
Ribo-seq reproducibility
0.987 0.981 0.984 0.991 0.980 0.991 0.988
0.988 0.991 0.975 0.989 0.976 0.990
0.998 0.984 0.996 0.985 0.994
0.984 0.999 0.986 0.997
0.981 0.998 0.988
0.983 0.996
0.990
WT-1 WT-2 elp3Δ ncs2Δ ncs6Δ-1 ncs6Δ-2 uba4Δ-1 uba4Δ-2
log2 RPKM
B
MSUM mutant comparisons: RNA-seq
0.493 0.175 0.534 0.211 0.444
0.240 0.587 0.285 0.582
0.193 0.679 0.236
0.238 0.570
0.259
elp3Δ ncs2Δ ncs6Δ-1 ncs6Δ-2 uba4Δ-1 uba4Δ-2
log2 fold change (mutant/WT)
C
MSUM mutant comparisons: Ribo-seq
0.623 0.405 0.614 0.401 0.543
0.495 0.743 0.459 0.628
0.505 0.740 0.515
0.528 0.664
0.557
elp3Δ ncs2Δ ncs6Δ-1 ncs6Δ-2 uba4Δ-1 uba4Δ-2
log2 fold change (mutant/WT)
D
ncs6Δ vs WT ΔRibo-seq RPKM
ncs6Δ vs WT ΔRNA-seq RPKM
r=0.64
all genes (5002)
GCN4 targets (393)
Ribo-seq changes (79)
overlap (24)
